# Mucopolysaccharidosis Type 1 among Children—Neuroradiological Perspective Based on Single Centre Experience and Literature Review

**DOI:** 10.3390/metabo13020209

**Published:** 2023-01-31

**Authors:** Magdalena Machnikowska-Sokołowska, Aleksandra Myszczuk, Emilia Wieszała, Dominika Wieja-Błach, Ewa Jamroz, Justyna Paprocka

**Affiliations:** 1 Department of Diagnostic Imaging, Radiology and Nuclear Medicine, Faculty of Medical Sciences in Katowice, Medical University of Silesia in Katowice, 40-547 Katowice, Poland; 2 Diagnostic Imaging and Interventional Radiology Centre, The Independent Public Clinical Hospital No 6, 40-752 Katowice, Poland; 3 Department of Pediatric Neurology, Faculty of Medical Sciences in Katowice, Medical University of Silesia in Katowice, 40-752 Katowice, Poland

**Keywords:** MPS 1, neuroradiology, mucopolysaccharidosis 1, Hurler disease, GAGs, glycosaminoglycans, magnetic resonance imaging

## Abstract

Mucopolysaccharidosis 1 (MPS 1) is a group of rare lysosomal genetic disorders resulting from the accumulation of undegraded glycosaminoglycans (GAGs) leading to multiorgan damage. Neurological symptoms vary from mild to severe. Neuroimaging—mainly magnetic resonance (MRI)—plays a crucial role in disease diagnosis and monitoring. Early diagnosis is of the utmost importance due to the necessity of an early therapy implementation. New imaging tools like MR spectroscopy (MRS), semiquantitative MRI analysis and applying scoring systems help substantially in MPS 1 surveillance. The presented analysis of neuroimaging manifestations is based on 5 children with MPS 1 and a literature review. The vigilance of the radiologist based on knowledge of neuroradiological patterns is highlighted.

## 1. Introduction

Mucopolysaccharidoses (MPS) are a group of inherited lysosomal storage disorders resulting from mutations of genes encoding various lysosomal hydrolases involved in degradation of glycosaminoglycans (GAGs, previously named mucopolysaccharides). These enzyme deficiencies lead to undegraded GAGs accumulation within the lysosomes, resulting in progressive cellular dysfunction and multisystemic disease. The MPS disorders are classified based on genes affected, lack or defect of specific lysosomal enzymes, age of presentation and typical clinical features [1].

MPS type 1 is an autosomal recessive metabolic disorder caused by mutations in alpha-L-iduronidase (IDUA) gene located on chromosome 4p16.3 [2]. These mutations lead to a deficiency of the glycosidase alpha-L-iduronidase (IDUA), which is required for the degradation of heparan and dermatan sulfates [3]. The prevalence of MPS 1 in the Polish population is approximately 0.22 cases per 100,000 births and accounts for 12% of all MPS cases. This is lower in comparison to data reported for other European countries [4,5]. A review of the available literature regarding MPS type 1 and its correlation to five own cases is the subject of this work.

## 2. Clinical Manifestations

Three major clinical subgroups are distinguished among Mucopolysaccharidosis type 1 (ICD-10 E76.0): Hurler (MPS-IH; OMIM #607014), Hurler-Scheie (MPS-IH/S; OMIM #607015) and Scheie (MPS-IS; OMIM #607016) phenotypes varying in age of onset, severity and rate of progression. However, this classification is not precisely delineated, neither clinically nor biochemically [6]. The clinical phenotype covers a broad spectrum of severity. Patients with severe, intermediate and mild features were historically classified as Hurler, Hurler-Scheie and Scheie syndromes, respectively. Hurler-Scheie and Scheie syndromes are referred to as “attenuated” or “non-Hurler” phenotypes [7]. Patients diagnosed with MPS usually do not manifest any characteristic features at birth.

Hurler syndrome presents in the most severely affected patients. Characteristic features include the onset of symptoms in early infancy, early progression of intellectual decline and death within the first decade of life if untreated [8,9,10]. Patients with Scheie syndrome reveal symptoms later in childhood and demonstrate less rapid progression with preserved cognitive functions and a lifespan reaching adulthood [11]. Hurler-Scheie is an intermediate form with none or mild central nervous system involvement, with patients usually surviving to adolescence or early adulthood when untreated.

Musculoskeletal manifestations are common features of all types of mucopolysaccharidoses and can be predominant among attenuated phenotypes. They typically include joint stiffness and contracture, carpal tunnel syndrome (normally uncommon in childhood), hip dysplasia, genu valgum, odontoid hypoplasia and dysostosis multiplex [12]. Dysostosis multiplex is the term used to describe the characteristic pattern of skeletal abnormalities in MPS patients. Among other clinical manifestations, the most characteristic ones include coarse facial features, a wide nasal bridge and a flattened midface that develop typically during the first year of life [13] along with a growth delay resulting in short stature [14]. Others include hepatosplenomegaly, umbilical and inguinal hernias, recurrent respiratory infections with chronic nasal discharge and frequent otitis media [15]. Development and growth peak during the first years of life, which are followed by the slowing down and evident decline further on [16]. Other characteristic features are the gradual loss of vision and hearing due to corneal clouding, optic nerve compression and combined conductive and sensorineural hearing loss. Cardiac abnormalities become evident up to the fifth year of life. These may include cardiomyopathy, endocardial fibroelastosis and valvular regurgitation, which can lead to heart failure [17]. Cutaneous lesions are not uncommon among patients, but they still remain very unspecific [18]. Characteristic clinical features of MPS 1 patients are summarised in Table 1.

The lifespan of Hurler syndrome patients is markedly shortened, on average not exceeding 10 years if untreated. Among individuals affected with Hurler-Scheie syndrome, death typically occurs in their twenties caused by cardiac or respiratory failure. Some Scheie syndrome patients can live over fifty years [5].

## 3. Differential Diagnosis Difficulties

An early diagnosis is crucial and known to improve outcomes for MPS patients by prompt treatment initiation with hematopoietic stem cell transplantation (HSCT) and enzyme replacement therapy (ERT) [15]. Supplementary management is based on relieving symptoms and supporting patients functioning. Among them, surgical interventions (neurosurgical, cardiosurgical, ENT and general surgery), physical therapies, hearing aids and medicine administration for pain and gastrointestinal disturbance management are listed [11,19].

Patients with MPS 1 display a wide range of clinical manifestations that vary in severity and the age of onset. The nonspecific nature of these symptoms results in significant diagnostic delays, particularly in patients with attenuated MPS 1 [20]. These patients with milder forms of the disease may in the first place seek medical attention because of musculoskeletal ailments that can easily be mistaken for inflammatory arthritides [12].

The clinical success of HSCT depends on the age of a child upon transplantation. If performed before developmental deterioration among MPS 1 patients, it can significantly preserve intellectual abilities and could improve or/and stabilise respiratory, hearing, vision, cardiac and articular function in patients as well. An early diagnosis is crucial for achieving the most effective treatment to different clinical manifestations of MPS 1 [21]. HSCT is proven as an effective treatment upon implementation according to the criteria of age (below 2 years old), developmental evaluation and prospective phenotype (DQ 70 or more) [22]. It is known to prevent cognitive and developmental deteriorations, diminish physical complications (hepatosplenomegaly, pulmonary, cardiovascular, hearing and vision loss) and elongate expected lifespan [21,23,24]. Unfortunately, psychosocial functional outcomes tend not to improve significantly, with numerous patients suffering from psychiatric disorders observed in long-term follow-up [24]. ERT, despite being implemented mainly in attenuated forms, usually does not meet with the desired effects [17]. Musculoskeletal MPS 1 manifestation alleviation is so far challenging, but new therapeutic options are emerging, including gene and anti-inflammatory therapies. Extensive research on possible treatment options was lately presented by independent teams [15,17]. Genttner et al. are currently conducting a clinical trial on autologous hematopoietic stem and progenitor cells (HSPC) transduced ex vivo transplantation combined with ERT. After a two year observation, their patients manifested the stabilisation of cognitive performance, motor skills and MRI findings, and they also presented reduced joint stiffness and followed regular growth progress (according to WHO Child Growth Standards) [25].

Newborn screening programs provide a good opportunity for an early diagnosis and treatment introduction, especially taking into consideration that most of the newborns present asymptomatic at birth [26]. There is a growing group of countries that perform newborn screening for MPS diseases. Three potential methods were proposed and implemented in early disease diagnosis, including a GAGs measurement, enzyme assay and molecular diagnosis [27]. The usual diagnostic path in high-risk patients starts with the measurement of urinary GAGs, which is followed by specific enzyme assays, to finally be concluded with the identification of a specific gene mutation.

## 4. Radiological Point of View

In general, the most frequent imaging findings among patients with MPS 1 are in the musculoskeletal system (e.g., thoracolumbar kyphosis) and central nervous system (most frequently white matter lesions, perivascular spaces (PVS) enlargement, brain atrophy and hydrocephalus).

Radiographic findings of dysostosis multiplex cover virtually the entire skeleton. Within the skull of people affected by the disease sella turcica is widened with flattening of its tuberculum, giving characteristic shape change (e.g., “J shape”). Dolichocephaly is a result of the premature closure of the sagittal suture. Macrocephaly, another characteristic feature of the disease, is typically caused by hydrocephalus and/or GAGs accumulation in the skull, meninges and brain. Additionally, the thickening of the calvarium is observed.

Spinal deformity and cord compression comprise another group of radiological findings caused by the involvement of the spine by MPS 1. Spinal canal stenosis leading to spinal cord compression originates from three major anomalies contributing to its development. These are: odontoid hypoplasia/aplasia, atlanto-axial instability/subluxation and thickening of periodontoid tissue, ligaments and duras that are resultant of GAGs deposition within surrounding soft tissues. Spinal cord compression, if untreated, may lead to its permanent damage. This can even be a life-threatening condition usually demonstrated as muscle strength lowering, tetraparesis and eventually death [28]. Another radiological feature characteristic of MPS is vertebral body deformities comprising hypoplastic anterosuperior areas of thoracolumbar transition vertebras with their anteroinferior beaking. along with dorsal kyphosis at this level, especially evident among the severe forms of MPS 1. Additionally to the listed bone anomalies, the pathology of the intervertebral discs might be observed in the form of their dehydration, decreased height and discs bulging [29]. Previously reported oral and dental findings of Hurler syndrome include macroglossia, high arched palate and hypoplastic teeth with their delayed eruption [30].

Hip dysplasia in MPS patients is common, generally progressive and results from poorly developed, flattened acetabulum, the underdevelopment of proximal femoral epiphysis and the coxa valga deformity (widened angle between the femoral neck and shaft) [31]. Flared iliac bones are another example of typical radiological finding of pelvic girdle. Long bones, in general, tend to be shorter and thicker with an irregular shaft and metaphysis as a result of their atypical remodelling. Within the distal parts of the forearm bones (ulna and radius), abnormal angulation and the tilting of distal epiphyses can be observed. Clavicles tend to have an altered shape with typically widened ends. In addition, ribs have a characteristic “oar shape” appearance, distinguished by the broadening of their anterior distal ends and narrowing of the opposite, paravertebral ends. That collectively results in reduced intercostal spaces—a thorax with a “beehive” appearance. Small bones are found with altered development as well, with proximally narrowed metacarpals and widening of their distal ends, both with irregular edges or “trigger digits”. Typical imaging findings of bony dysplasia in MPS are summarised in Table 2.

## 5. Neuroimaging

Among patients with Hurler disease, the clinical picture is usually dominated by the involvement of the central nervous system, including mental retardation [16]. Neuroimaging studies in that group of patients are performed most frequently, usually upon the suspicion of indirect CNS damage due to hydrocephalus, craniocervical instability or spinal cord compression.

Imaging plays a crucial role in the early suspicion and monitoring of disease progression, especially craniocervical junction stenosis, spinal cord compression and hydrocephalus. An early recognition allows prompt intervention, preventing permanent damage [32].

Contrast-enhanced magnetic resonance is a gold standard in central nervous system imaging. White matter signal (WMS) abnormalities are frequent findings on a brain MRI, appearing as focal or diffuse, T2-hyperintense, typically with periventricular and bilateral arrangement. WMS abnormalities, if reported early, have a tendency to expand on subsequent examinations with patients’ growth. These abnormalities may originate from: (1) delayed myelination, (2) demyelination or (3) gliosis. Enlarged PVS are isointense to cerebrospinal fluid (CSF) and were reported within the white matter (WM), corpus callosum (CC), basal ganglia and brainstem. Among these, the involvement of CC appears to be the most distinctive upon comparison of MPS 1 patients and healthy controls [33]. A reduced CC volume had been reported among Hurler syndrome patients, standing out from attenuated phenotypes, and even more markedly from the healthy control group [34]. There even was a suggestion of correlation between smaller CC volume and decreased attention [35]. Another unique imaging finding of MPS is the “honeycomb-like” appearance of the basal ganglia and thalami. Hurler syndrome patients have also been described having abnormalities of the posterior cranial fossa. The most common of these findings were mega cisterna magna and white matter changes in the cerebellum [29]. An optic nerve sheath enlargement was a relatively rare finding among MPS 1 patients, appearing more frequently among other mucopolysaccharidosis types.

**Scheme 1:**
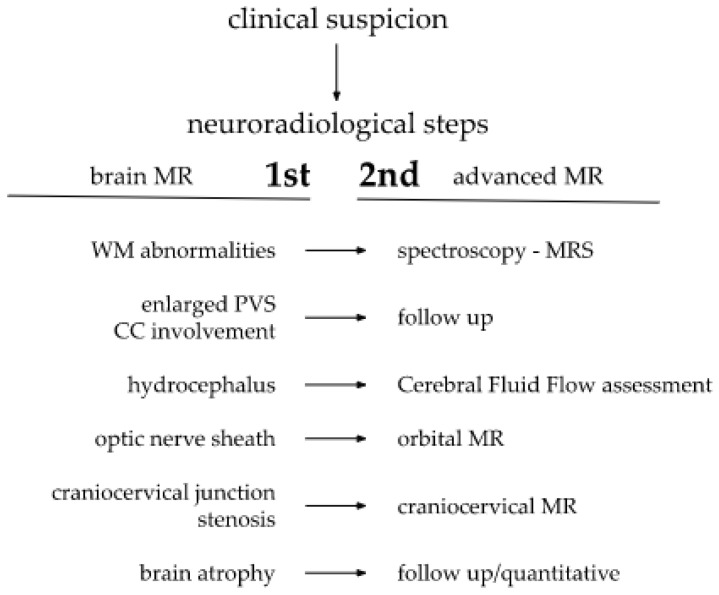
Proposed steps of MR neuroimaging.

Depending on alterations observed by the radiologist during a brain MRI, a decision to extend the current protocol with additional sequences, as well as the need to schedule additional advanced imaging, could arise. Upon the detection of white matter abnormalities, a brain metabolites concentration analysis in magnetic resonance spectroscopy (MRS) would be adequate. Some authors even propose MRS as an important tool for neurological processes and cognitive functions evaluation [36]. Proposed steps of MR neuroimaging are presented on a Scheme 1.

If hydrocephalus is present, the cerebrospinal fluid (CSF) flow could be assessed in supplementary, dedicated sequences. In turn, a focused orbital MRI could be of particular use for the optic nerve sheath enlargement precise measurement. Distortions within the craniocervical junction, which is characteristic for MPS patients, frequently lead to spinal canal stenosis and respiratory tract narrowing [29]. This could subsequently lead to future potential intubation difficulties. Therefore, the early imaging of the craniocervical junction is helpful in the planning proper timing of surgical interventions, crucial for overtaking neurologic and anesthesiologic complications.

Delayed neurodevelopment may additionally be amplified by hearing loss, associated with fluid accumulation in the temporal bone and eustachian tube closure, a result of GAGs accumulation. These can easily be noticed in a head MRI. Summarised imaging features of CNS are presented in Table 3.

## 6. Patients Presentation

**Figure 1:**
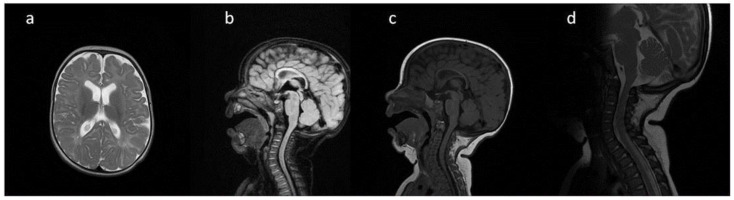
Brain and cervical spine MRI. Patient 1. (younger sister) (**a**) axial T2; (**b**) sagittal T2 FLAIR, (**c**) sagittal T1; (**d**) sagittal spinal T2. Findings: delayed myelination, slight skiboot sella turcica shape; suspicion of dens hypoplasia—for observation (age 4 mo).

**Figure 2:**
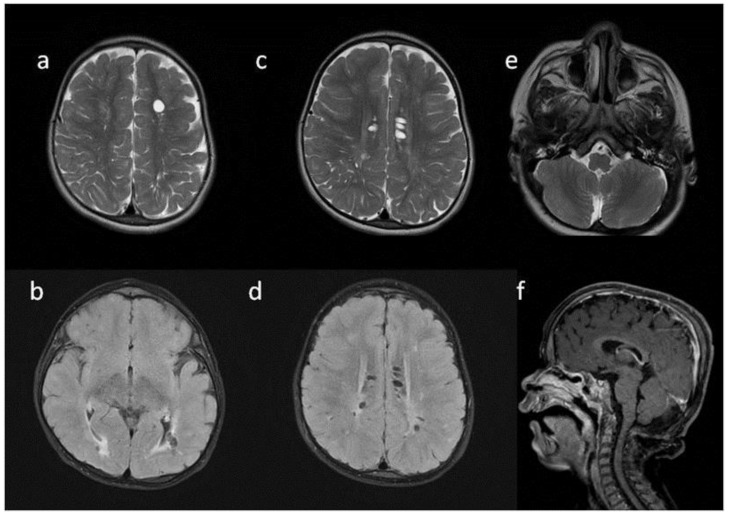
Brain MRI. Patient 2. (**a**,**c**,**e**) axial T2; (**b**,**d**) axial T2 FLAIR; (**f**) sagittal T1. Findings: (**a**,**c**,**d**) enlarged PVS with characteristic location in corpus callosum; (**a**–**d**) abnormal white matter signals; (**e**) posterior fossa horns; (**f**) J-shaped sella turcica, dysplastic dens.

**Figure 3:**
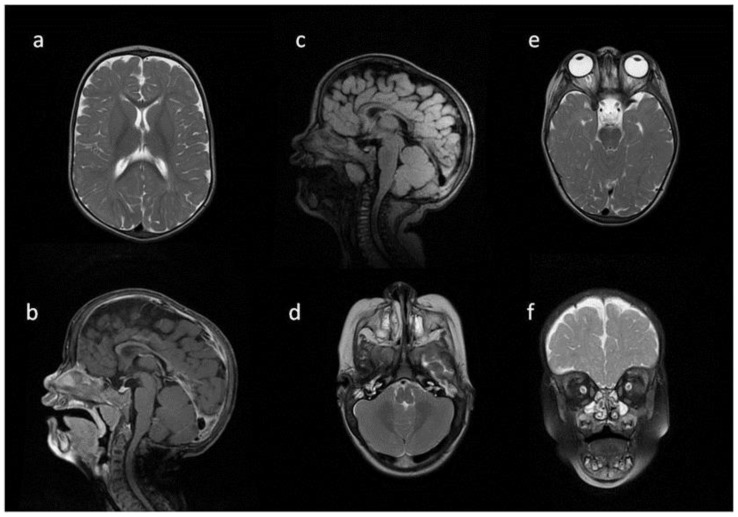
Brain MRI. Patient 3. (**a**,**e**,**d**) T2 axial; (**f**) T2 coronal; (**b**,**c**) T2 sagittal; (**b**) T1 contrast-enhanced; (**c**) T2 FLAIR. Findings: (**a**) delayed myelination; (**b**,**c**) J-shaped sella turcica, dysplastic dens; (**d**) temporal bone air cells effusion, posterior fossa horns; (**e**,**f**) enlarged optic nerve sheath.

**Figure 4:**
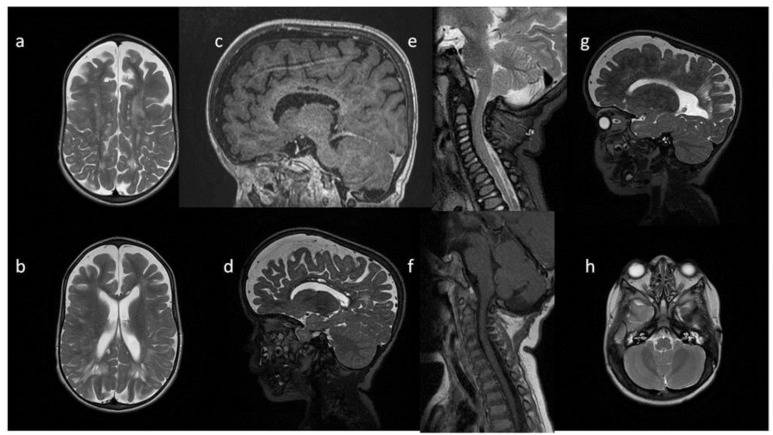
Brain MRI. Patient 4, 1st exam. (**a**–**d**) axial T1 & T2; (**e**–**h**) sagittal T1, T2, Flair T2, T1. Findings: (**a**,**c**,**g**) enlarged periventricular spaces with characteristic location in corpus callosum; (**a**,**c**,**g**) abnormal white matter signals; (**b**) posterior fossa horns, mastoid cells fluid; (**d**) optic nerve sheath enlargement, ethmoid sinus fluid; (**e**,**f**,**h**) J-shaped sella turcica, dysplastic dens; (**e**,**g**) skull deformation.

**Figure 5:**
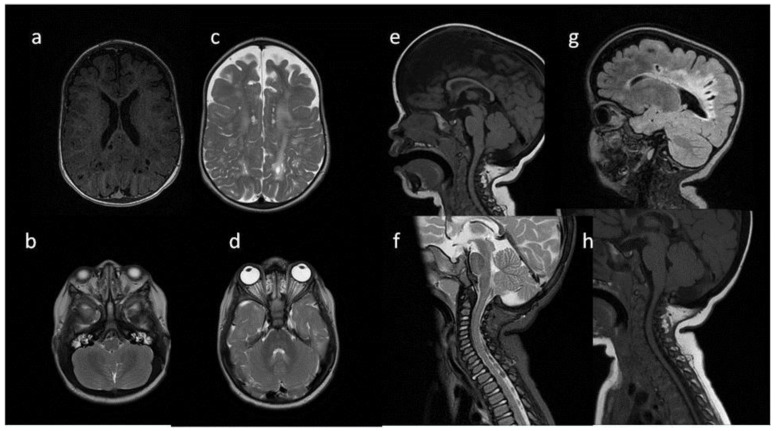
Brain and cervical spine MRI. Patient 4, follow up: (**a**,**b**,**h**) axial T2; (**c**,**d**,**g**); sagittal brain (**c**) T1; (**d**,**g**) T2; (**e**,**f**) sagittal spine (**e**) T2, (**f**) T1. Findings: (**a**–**d**) enlarged PVS with characteristic location in corpus callosum; (**a**,**b**,**d**,**g**) abnormal white matter signals;(**e**,**f**) vertebral spaces and bodies deformity, J-shaped sella turcica, dysplastic dens, (**h**) mastoid cells fluid, discrete posterior fossa horns, (**a**–**d**,**g**) skull deformity and enlarged frontal subarachnoid spaces.

**Figure 6:**
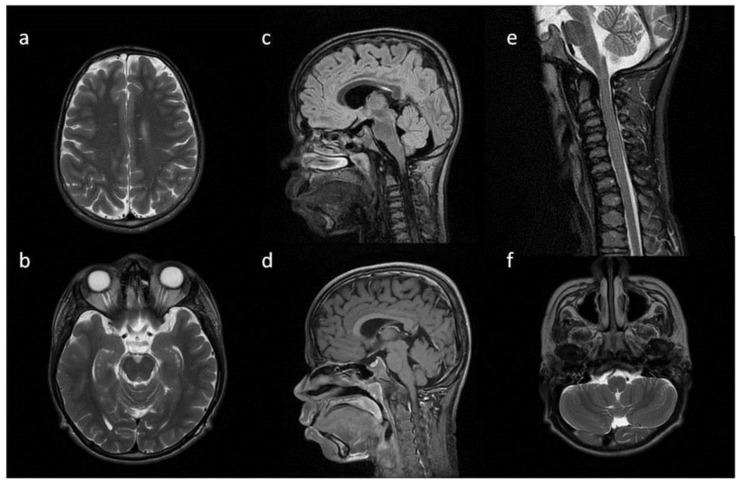
Brain and spine MRI. Patient 5. (**a**,**b**,**f**) axial T2; (**c**,**d**) sagittal brain; (**c**) T2 FLAIR; (**d**,**g**) T1; (**e**) sagittal spine; (**e**) T2; Findings: (**a**) enlarged PVS with characteristic location in corpus callosum; (**b**) optic nerve sheath enlargement; (**c**,**d**) flattened anterior border of sella turcica, dysplastic dens; (**f**) mastoid cells fluid; (**e**) vertebral and intervertebral discs deformation, cervical spine kyphosis and stenosis.

We present cases of five children diagnosed with MPS 1, Hurler type. Taking into consideration the rarity of the condition, our group seems significant. Their clinical features and analysis of neuroradiological symptoms are summarised in two following tables (Table 4 and Table 5) and figures (Figure 1, Figure 2, Figure 3, Figure 4, Figure 5 and Figure 6).

## 7. Neuroimaging—Methods

Brain and spine MRI were performed using Optima 1.5 T or Artist 1.5 T GEM (General Electric, GE Healthcare, Milwaukee, WI, USA). The imaging protocol of brain MRI included the standard sequences: T1, T2, T2 Flair, DWI (diffusion weighted imaging) and SWI (susceptibility weighted imaging) and T1 3D post contrast. The imaging protocol of spine MRI included the standard sequences: sagittal T1, T2, STIR, DWI, 3D CUBE T2, when scoliosis is present—coronal 3D STIR. For the assessment of cranio-cervical junction focused 3D T1 or T2 sequence is additionally performed. The dose of gadolinium contrast is 0.1 mL/kg of the body weight. The data are transferred to a commercially available Workstation ADW 3.2. A head CT had limited clinical use, only in craniosynostosis in children with MPS, due to a lack of sensitivity in the brain assessment, additionally posing the danger of radiation, against the ALARA (as low as reasonably achievable) dose rule.

## 8. Brain, Head and Spine—Radiological Symptoms

The most consistent findings among our patients were observed at the border of the head MRI. All five of them presented with sella turcica distortions and fluid effusion in the temporal bone. Within the brain, either delayed myelination or white matter (WM) signal abnormalities were observed. Although two of the examined children did not present PVS enlargement, one of the remaining three cases had typical corpus callosum PVS enlargement. Other symptoms are listed in the Table 5 below.

**Table 5:** Brain, head and spine. Radiological symptoms.

Patient No	1	2	3	4		5
age at exam	4 mo	3 yo	1 y 2 mo	1 y 11 mo	3 y 2 mo	14 yo
radiological imaging						
WM signal abnormalities		+		+	+	
areas of delayed myelination	+	+	+	+	+	
enlarged perivascular spaces		+		+	+	+
narrow corpus callosum		+		+/−	+	+
prominent ventricular system	+/−	+		+/−	+	+
arachnoid cyst				+	+	
optic nerve sheath enlargement			+		+	
J-shaped sella turcica	+	+	+	+/−	+	+/−
craniocervical junction distortion		+	+/−		+	+
posterior fossa horns		+	+		+	+
fluid effusion temporal bone	+	+	+		+	+/−
closed sagittal suture		+		+	+	
vertebral bodies deformity					+	+
intervertebral disc anomalies			+			+

## 9. Discussion

Raising awareness of MPS 1 among physicians and radiologists is of crucial importance for the proficient recognition of typical clinical manifestations and imaging findings of the disease in its early stages. It could elicit an early extension of diagnostics and prompt treatment implementation [37].

After the initial clinical evaluation, children with existing stature and growth anomalies are usually referred for X-ray for precise evaluation of bone anomalies. Children with attenuated forms of MPS may start to seek medical advice only after puberty. Suspicion is often initially raised by a rheumatologist or orthopedist upon joint and bone abnormalities detection [20]. In 2022, an algorithm for children with growth abnormalities dedicated mainly to pediatricians and endocrinologists was proposed by an Italian multidisciplinary group. It aimed at improving the clinical awareness of MPS 1, especially its attenuated forms. The mentioned algorithm was established on the basis of clinical and radiological features accompanying growth retardation [38]. These children are reviewed by general and pediatric radiologists most of the time; therefore, drawing their attention to distinctive skeletal abnormalities is necessary.

Among the youngest patients, before the onset of symptoms, the only possibility for early diagnosis and treatment is adequate metabolic screening [10,26]. Unfortunately, even though pilot studies have been conducted in a few countries, the low accessibility and high costs of the possible methods diminish the probability of implementing worldwide MPS screening [26,39].

In 2018, a group of 11 experts in pediatric metabolic diseases proposed an algorithm for prompt MPS 1 diagnosis, which was tested on 35 patients [10]. They stated that 91% of assessed patients would have been referred for earlier for diagnosis and treatment if the tool was implemented. It is based on multiorgan symptoms with the apparent domination of musculoskeletal pathologies [10]. These patients who have undergone scrutinised clinical assessment report to the MRI already with a suspicion of MPS.

A different group consisted of children diagnosed due to developmental delay. This feature is very unspecific and, in MPS 1, found early only in children with Hurler disease. In our opinion, an early referral for brain MRI among children with developmental delay might become another key instrument for raising a strong suspicion of MPS 1. Neuroimaging in children with non-characteristic features may arise an early suspicion of MPS only with the awareness of a reporting radiologist.

Imaging difficulties lie in unspecific signs, existing in multiple other degenerative neurological diseases, both progressing and stable. Recently published papers confirm our hypothesis, emphasising the significance of neuroradiological findings and their importance during therapy selection [23,32].

Prominent or enlarged PVS—either cribriform or sieve-like—seem to be one of the most important features in the brains of MPS patients, appearing in young patients. Marked PVS must be differentiated with multiple anatomical variants and pathological states, as they could manifest in numerous other microvascular, neurometabolic, inflammatory and neoplastic diseases or have a traumatic origin. According to the literature, the PVS enlargement that is located within the CC seems to be the most distinctive for patients with MPS [40]. Some authors reported an unusual location of prominent PVS: peripherally at the cortical junction, naming it a “pearl-of strings pattern” [32]. In contrast to most authors, prominent PVS were found among three of our patients, with only one having the typical localisation. This might be compatible with the concept of disturbed CSF resorption resulting from GAG deposits in the leptomeninges and astrocytes [41].

The myelination process of white matter in MPS children tends to be delayed. Additionally, patchy white matter T2 hyperintensities have been reported [42]. At the early stages of development, a solely delayed myelination may be evident, which could be subsequently transformed into abnormal hyperintense signals within WM. The accumulation of GAGs in neurons and astrocytes is suspected to cause neuronal volume loss and the brain MRI signal abnormalities that follow [29,32]. This was the most consistent finding detected in all five of our children.

In 2022, Kovac et al. [34] published the results of a multicenter study on the quantitative brain morphology of MPS 1 severe and attenuated forms compared to a control group. They found a correlation between the volumes of grey and white matter, CC and ventricles and age. The implementation of these measurements might contribute to further MRI application for observing age-related brain changes and also help with establishing a correlation between neurocognitive function, brain structure alterations and treatment monitoring (HSCT). Similarly, a positive association between CC volumes and attention in Hurler disease patients, in comparison to attenuated forms, was described by King et al. in a volumetric MRI [35].

Identical mechanisms of GAGs accumulation are suspected to cause hydrocephalus and cerebral atrophy (manifested as subarachnoid spaces enlargement) [43]. All of our patients had a varying degree of enlargement of the lateral and/or third ventricles. In turn, meningeal accumulation of GAGs is believed to cause arachnoid cysts formation described in the literature [42]. Two of our patients manifested with arachnoid cysts at the temporal lobes.

Concurrently with diagnosing the brain pathology, possible skull and facial abnormalities at the borders of a brain MRI cannot be omitted. Various shape alterations of sella turcica have been reported, namely J-shaped [44], skiboot [45], omega [44] and sellas, with possible coexistence of an empty sella, both enlarged or diminished in size. Three of our patients had J-shaped sella turcica. The remaining two were reported with flattening of anterior sellar border and slightly expressed the skiboot shape, as they were the oldest and the youngest of the observed group, respectively. In 2019 Damar et al. reported the occurrence of J-shaped sella in 100% of their Hurler patients, and what is more, most of them had the coexistence of tympanic effusion. They also have described an original finding—internal hypertrophy of the occipitomastoid suture (IHOMS). Other cranial deformities were also noticed. Having analysed a complex group of MPS patients, they concluded that cranial pathologies might help in differentiating MPS types [33]. Similarly to Damar, IHOMS of varying expression degree were detected in all five of our children. Differential diagnosis of sellar anomalies must comprise anatomical variants, achondroplasia, osteogenesis imperfecta, chronic hydrocephalus, optic chiasm glioma and neurofibromatosis type 1.

One of the most debilitating effects of enzyme insufficiency in MPS 1 children, mainly with Hurler disease, are craniocervical junction distortions. Only one child from our group did not manifest this feature—the youngest girl, who was only 4 months old upon Hurler disease diagnosis, ahead of clinical symptoms manifestation. Such an early diagnosis was possible only because of the previous confirmation of the disease in the older sister. CC junction abnormalities arise mostly as a result of different stages of dens underdevelopment (from total aplasia, through hypoplasia, to normal length). A laxity of periaxial ligaments adds to the risk of acute/chronic injury of the spinal cord [32,42]. Miebach et al. [46] analysed the available data on the treatment response of the craniocervical junction to HSCT in Hurler disease. They again emphasised that the implementation of a proper neuroimaging MRI protocol in a possibly vast group of post-HSCT patients could contribute to objective measurements for evaluating CC junction response to treatment. All but one of our patients manifested with varying degrees of dens hypoplasia or basilar invagination.

Aside from CC junction abnormalities, spinal problems include spinal stenosis, kyphosis and resultant compressive myelopathy. An MRI is irreplaceable in the precise visualisation of the spine and spinal cord abnormalities.

Another typical cranial manifestation of MPS 1 is craniosynostosis. Simultaneously with neurological problems, it can contribute to optic nerve canal narrowing and increased pressure in the optic nerve sheath. The accumulation of GAGs in the sheath itself contributes to its widening. The final result could be optic nerve atrophy—a common MPS 1 complication [29,47].

GAGs accumulation also potentially gives rise to ENT and airway problems on all levels, with resultant breathing and hearing complications. Tonsillar and lingual enlargement, mucosal thickening and increased mucus production induce fluid effusion in air spaces (sinuses and petrous bones). Fluid effusion of the temporal bone was reported in all our patients.

MRS is a method used for the identification of different metabolites in the brain. Takahashi et al. measured the resonance of chemical substances in the brain and urine of MPS 1 Hurler patients. They identified a resonance peak at 3.7 ppm, slightly higher than the shift of myoinositol. Additionally, they stated that a diminished choline/creatine ratio could be a presumptive mucopolysaccharide (pMPS) and proposed MRS with the evaluation of choline, N-acetylaspartate (NAA) and pMPS as an important objective tool in the diagnosis and treatment monitoring of MPS 1 patients [36].

A multicentre study by Pontesilli et al. proposing a new MRI scoring system was published in 2022. Based on review of nine Hurler syndrome patients post-HSCT, they stated that the proposed scoring system based on brain and scale MRI correlates with a cognitive outcome and clinical prognosis [23]. With the emergence of new methods of MPS 1 treatment [25], tools for objective, quantitative or semiquantitative measurements of the brain and spine would be helpful more than ever. Classical and advanced protocols of MRI, therefore, play a crucial role in the primary diagnosis, observation, and, most importantly, in monitoring disease progression in patients (i.e., for the proper timing of CC junction surgery and the assessment of the response to the applied therapies).

## 10. Conclusions

A diagnosis of MPS 1 starts with an alert pediatrician and neurologist. Upon suspicion of the diagnosis, a wide range of diagnostic procedures should be implemented. Despite neuroimaging features being neither pathognomonic nor very characteristic for MPS 1, a vigilant neuro/pediatric radiologist reporting them on the brain MRI might direct the diagnostic path accurately. The correlation of PVS, WMA, hydrocephalus and other described anomalies with the severity of the disease (especially with the developmental delay) remains controversial. So far, published studies have not yet proposed an unequivocal imaging method that could substitute conventional clinical and biochemical markers of disease severity. A few scoring systems were proposed by groups of researchers during recent years as an approach for increasing the importance of imaging. At the present day, radiology plays a crucial role in monitoring craniocervical junction and spinal stenosis, myelopathy, hydrocephalus and PVS enlargement.

Further methodical, multicenter studies based on large research groups of MPS 1 patients with the implementation of a unified imaging protocol could substantially expand the knowledge of the pathogenesis and aid in the evaluation of responses to possibly emerging therapies.

## Figures and Tables

**Table 1:** Characteristic clinical MPS 1 manifestations.

System Affected	Problem
General appearance	coarse facies short stature
Musculoskeletal	dysostosis multiplex gibbus kyphosis scoliosis hip dysplasia genu valgum joint stiffness and contractures odontoid hypoplasia
Neurological	cognitive impairment hydrocephalus carpal tunnel hearing loss
Respiratory/ENT	recurrent respiratory infections otitis media
Cardiovascular	valvular disease heart failure arrhythmias
Ophthalmology	corneal clouding glaucoma retinal changes
Gastrointestinal	hepatomegaly splenomegaly inguinal/umbilical hernia IBS/diarrhoea *
Skin lesions (non-specific)	thickened skin excessive hair growth extensive melanocytosis

* especially among MPS-IH post HSCT.

**Table 2:** Typical imaging findings of bony dysplasia in MPS.

Dysostosis Multiplex	
skull	shape changes of sella turcica craniosynostosis macrocephaly thickened calvarium oral and dental abnormalities
spine	craniocervical junction anomalies cord compression spinal deformities
other bones	inadequate modelling of long bones clavicles with widened ends “oar shaped” ribs metacarpals deformation flared iliac bones with flattened acetabulum bilateral coxa valga deformity increased risk of osteoporosis/osteopenia

**Table 3:** CNS imaging features.

Neuroimaging Findings—Main Changes
white matter signal abnormalities enlarged perivascular spaces brain atrophy hydrocephalus spinal canal stenosis involvement of corpus callosum

**Table 4:** Clinical analysis of children’s symptoms.

Patient No	1	2	3	4	5
diagnosis age current age sex	4 mo 8 mo F	2 y 10 mo 10 y 5 mo F	1 y 2 mo 1 y 3 mo F	2 y 4 mo 3 y 10 mo F	1 y 10 mo 15 y 5 mo M
clinical features					
level of urinary GAGs at diagnosis	462 mg/g creatinine (N: 90.0–208.0)	459.95 mg/g creatinine (N: 90.0–208.0)	179.2 mg/mmol creatinine (N: 9.52–26.9)	745 mg/g creatinine (N: 90.0–208.0)	509 mg/g creatinine (N: 83.0–161.0)
alpha-L-iduro- nidase activity at diagnosis	0 umol/L/h * (cut-off value > 1.5)	0.01 nmol/mg protein/18 h ** (N: 88.5–169.5)	0.2 umol/L/h * (cut-off value > 1.5)	0.3 umol/L/h * (cut-off value > 1.5)	0.01 nmol/mg protein/18 h ** (N: 88.5–169.5)
mutations in the alpha-L-iduronidase gene	p.Gln63Ter and p.Arg621Ter	p.Gln63Ter and p.Arg621Ter	in progress	homozygous c.1045_1047delGAC/p.(Asp349del)	not done
family history	MPS type 1 in older sister	MPS type 1 in younger sister	unencumbered	suspicion of multiple sclerosis in father	unencumbered
developmental stages at psychomotor skills	wide range (asymmetrical body muscle tone)	correct physical and delayed mental and speech development	delayed (sitting from 11 mo, does not walk alone, words 13 mo)	delayed (walk from 2 y 4 mo, words 12 mo, halted speech development)	wide range (walk from 15 mo, words 12 mo, sentences 3 yo)
treatment	ERT since 5 mo old, qualified for HSCT	ERT 3 to 5 yo; HSCT at 4 y 4 mo	qualified for ERT	ERT since 2 y 6 mo old, qualified for HSCT	ERT since 2 years old until now

* DBS (dried blood test), ** (activity in leukocytes).

## Data Availability

The data presented in this study are available on request from the corresponding author. The data are not publicly available due to ethical reasons.

## Abbreviations

CC	corpus callosum
CSF	cerebrospinal fluid
DQ	developmental quotient
ERT	enzyme replacement therapy
GAGs	glycosaminoglycans
HSCT	hematopoietic stem cell transplantation
HSPC	hematopoietic stem and progenitor cell
IDUA	alpha-L-iduronidase
IHOMS	internal hypertrophy of the occipitomastoid suture
MPS 1	Mucopolysaccharidosis type 1
MRI	magnetic resonance imaging
MRS	magnetic resonance spectroscopy
PVS	perivascular spaces
WM	white matter
WMA	white matter abnormalities
